# Molecular Pathways and Respiratory Involvement in Lysosomal Storage Diseases

**DOI:** 10.3390/ijms20020327

**Published:** 2019-01-15

**Authors:** Paola Faverio, Anna Stainer, Federica De Giacomi, Serena Gasperini, Serena Motta, Francesco Canonico, Federico Pieruzzi, Anna Monzani, Alberto Pesci, Andrea Biondi

**Affiliations:** 1 Department of Medicine and Surgery, Università degli Studi di Milano Bicocca, Respiratory Unit, San Gerardo Hospital, ASST di Monza, 20900 Monza, Italy; annetta.stainer@gmail.com (A.S.); i.fede@live.it (F.D.G.); annamonz75@gmail.com (A.M.); alberto.pesci@unimib.it (A.P.); 2 Rare Metabolic Diseases Unit, Pediatric Department, Fondazione MBBM, Università degli Studi di Milano Bicocca, San Gerardo Hospital, ASST di Monza, 20900 Monza, Italy; serena.gasperini69@gmail.com (S.G.); sere.motta@libero.it (S.M.); andrea.biondi@unimib.it (A.B.); 3 Department of Neuroradiology, Università degli Studi di Milano Bicocca, San Gerardo Hospital, ASST di Monza, 20900 Monza, Italy; f.canonico@asst-monza.it; 4 Department of Medicine and Surgery, Università degli Studi di Milano Bicocca, Nephrology Unit, San Gerardo Hospital, ASST di Monza, 20900 Monza, Italy; federico.pieruzzi@unimib.it

**Keywords:** lysosomal storage diseases, lung involvement, mucopolysaccharidosis, mucolipidoses, Pompe disease, Niemann-Pick disease, Gaucher’s disease, Fabry disease

## Abstract

Lysosomal storage diseases (LSD) include a wide range of different disorders with variable degrees of respiratory system involvement. The purpose of this narrative review is to treat the different types of respiratory manifestations in LSD, with particular attention being paid to the main molecular pathways known so far to be involved in the pathogenesis of the disease. A literature search was conducted using the Medline/PubMed and EMBASE databases to identify studies, from 1968 through to November 2018, that investigated the respiratory manifestations and molecular pathways affected in LSD. Pulmonary involvement includes interstitial lung disease in Gaucher’s disease and Niemann-Pick disease, obstructive airway disease in Fabry disease and ventilatory disorders with chronic respiratory failure in Pompe disease due to diaphragmatic and abdominal wall muscle weakness. In mucopolysaccharidosis and mucolipidoses, respiratory symptoms usually manifest early in life and are secondary to anatomical malformations, particularly of the trachea and chest wall, and to accumulation of glycosaminoglycans in the upper and lower airways, causing, for example, obstructive sleep apnea syndrome. Although the molecular pathways involved vary, ranging from lipid to glycogen and glycosaminoglycans accumulation, some clinical manifestations and therapeutic approaches are common among diseases, suggesting that lysosomal storage and subsequent cellular toxicity are the common endpoints.

## 1. Introduction

**Figure 1:**
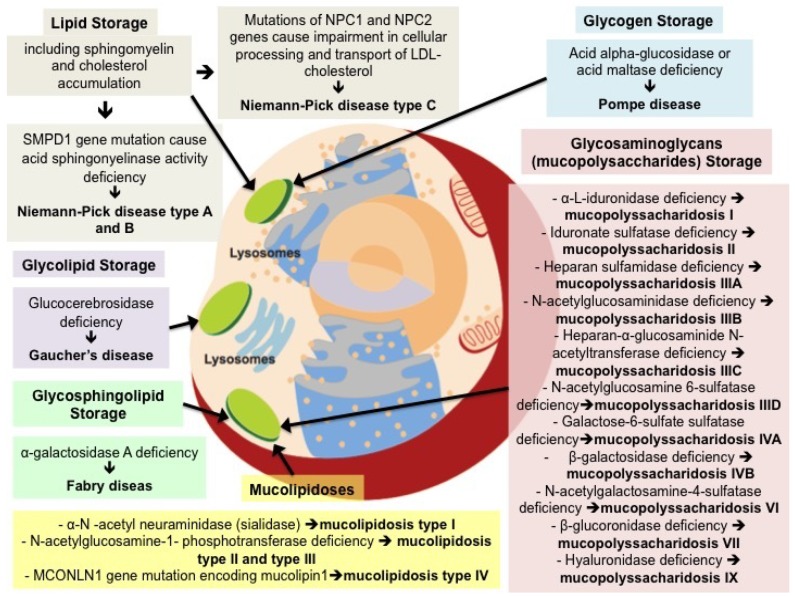
Molecular pathways involved in lysosomal storage diseases. LDL = Low-density lipoprotein.

Lysosomal storage diseases (LSD) covers a wide range of disorders with different underlying molecular pathways, Figure 1.

**Figure 2:**
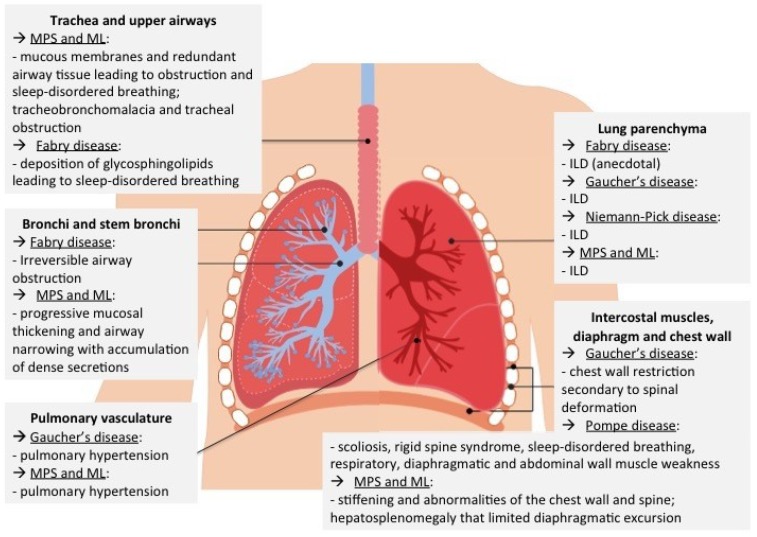
Respiratory system involvement in different lysosomal storage diseases. MPS = Mucopolysaccharidosis, ML = Mucolipidoses, ILD = Interstitial Lung Disease.

The respiratory system may present variable degrees of involvement and clinical scenarios, Figure 2.

Possible manifestations range from interstitial lung disease in Gaucher’s disease and Niemann-Pick disease, through obstructive ventilatory disorders in Fabry Disease, to chronic respiratory failure secondary to muscle weakness in Pompe disease and holistic (both obstructive and restrictive ventilatory) impairment in mucopolysaccharidosis (MPS) and mucolipidosis (ML), Figure 2.

Multiple therapeutic approaches are available, from disease-specific enzyme replacement therapy (ERT) and substrate reduction therapy (SRT) to treatment of the various clinical manifestations, including non-invasive ventilation (NIV) for obstructive sleep apneas (OSA) and tracheal (-vascular) reconstructive surgery in severe anatomical malformations and redundant airway tissue.

The purpose of the present narrative review is to treat the different types of respiratory involvement in LSD, with particular attention being paid to the main molecular pathways known so far to be involved in the pathogenesis of the disease.

A search of relevant medical literature was conducted in Medline/PubMed including observational and interventional studies from 1968 through 2018. Editorials, narratives and conference abstracts have been excluded. Keywords used to perform the research are reported in Table 1.

## 2. Mucopolysaccharidosis and Mucolipidoses

MPS are a group of rare lysosomal disorders characterized by deficiencies in several enzymes that catalyze the degradation of different glycosaminoglycans (GAG). MPS are characterized by a progressive course with multisystemic involvement and are split into 7 distinct types (I, II, III, IV, VI, VII and IX), Figure 1. Each form presents a wide spectrum of clinical severity: i.e., in MPS I, the severe form is call Hurler, the intermediate is Hurler-Scheie and the attenuated form is Scheie syndrome. All the defects have autosomal recessive inheritance except for MPS II (Hunter disease), which has a X-linked means [1]. The overall incidence of MPS is estimated to be as high as 1:25,000 births [1].

ML are a group of autosomal recessive diseases mainly due to storage of oligosaccharides with features overlapping MPS: ML I (sialidosis), ML II (a severe form also called I-cell disease), ML III (pseudo-Hurler polydistrophy) and ML IV, Figure 1.

MPS and ML are variably characterized by progressive mucosal thickening, airway narrowing, and gradual stiffening of the chest wall until restrictive respiratory impairment [2].

### 2.1. Type of Respiratory Involvement

Impairment in pulmonary function is an important and frequent problem for MPS and ML patients: upper and lower airways of these patients are often early affected in the disease course even in attenuated forms [3,4]. Respiratory manifestations are mainly related to GAG/oligosaccharides storage, which initially causes obstruction in the upper airways. Tracheobronchial symptoms such as tirage and cornage occur later, and are frequently the cause of death. Restrictive lung disease is commonly observed in patients with significant skeletal involvement, especially MPS IV (Morquio) and MPS VI (Maroteaux-Lamy). Other common manifestations are rhinosinusitis, chronic otitis media, accumulation of dense secretions in the upper and lower airways causing frequent infections, hearing impairment, noisy breathing and sleep apneas. MPS patients typically undergo multiple surgical procedures even before diagnosis, despite being at increased risk for anesthetic complications [5].

All patients affected by MPS I, particularly the Hurler phenotype (the severe form), are at risk of severe respiratory failure as a result of restrictive lung disease, OSA, and/or asthma [6]. Only MPS III (Sanfilippo) shows less respiratory involvement, because heparan sulphate storage mainly involves brain.

Anatomical malformations of the face, such as a coarse face with prominent cheek bones, short neck, plump lips, macroglossia and flattened nasal bridge may cause ventilatory impairment. Similarly, anatomical alterations of the upper airways, such as upper airway narrowing by enlarged tonsils, adenoids, mucous membranes and redundant airway tissue, also lead to higher airways resistance and pharyngeal collapse with stridor and OSA [7,8].

Thickened vocal cords with alterations of mucosa of the larynx and hypopharynx is more evident in MPS II; redundant mucosa relapsing into the larynx and moving with the airflow causes noisy breathing [9].

Tracheobronchomalacia and tracheal obstruction that ultimately lead to respiratory failure due to GAG/oligosaccharides accumulation in chondrocytes and extracellular matrix of trachea are also common [9]. Another hypothesis is the imbalanced growth of the trachea and vessels in comparison with cervicothoracic spine and ribs. According to Kubaski et al., 67.9% of patients affected by MPS IVA seem to have a triangular-shaped tracheal narrowing associated with increased tracheal tortuosity that worsened with age [10]. The tracheal narrowing can also be attributed to compression by the tortuous brachiocephalic artery and can be evident as early as the age of 2 years. Tracheostomy can be difficult in MPS IVA patients due to tortuous and redundant trachea, short neck, and inability to hyperextend the neck. Pizarro et al. described a new surgical procedure consisting of a tracheal vascular reconstruction without tracheostomy that should improve tracheal obstruction [11,12,13]. With progressive tracheomalacia, a regular tracheostomy tube may also be insufficient, and longer and wider tubes might be necessary; however, this kind of tube can cause deposition of granulation tissue and further accumulation of GA [5,14]. In MPS II, tracheal and bronchial collapse have also been described. They result in obstructive airway disease, leading to trapped air that, combined with restrictive lung disease, can lead to respiratory failure, particularly during acute airway infections [15].

Pulmonary function tests (PFTs) often reveal restrictive lung disease, which is predominantly due to extrinsic causes: a combination of skeletal abnormalities of the chest and spine, including decreased thoracic dimensions due to short stature, scoliosis and thoracic deformities (hyperkyphosis, pectus carenatum, cardiomegaly), and reduced diaphragmatic excursion due to lumbar hyperlordosis, gibbus and hepatosplenomegaly [3,7]. Other infrequent causes of restrictive impairment are alveolar and interstitial pulmonary involvement with pulmonary hypertension [4].

Sleep-disordered breathing (SDB) is also frequently observed in MPS and ML patients; obstructive and central sleep apneas are more frequent during rapid eye movement (REM) sleep because of the reduced tone of accessory respiratory muscles and decreased ventilatory carbon dioxide chemosensitivity during this sleep phase. Central apnea is due to several factors in MPS/ML patients, including spinal cord compression, increased intracranial pressure causing compression of the sleep regulatory centers in the brainstem, neurotransmitters alterations, abnormal sleep cycle [16]. The prevalence of OSA syndrome in MPS patients is at least 40% (4 times greater than the general population), and these patients seem to be more susceptible to OSA-induced hypoxemia [17]. Anatomical abnormalities may also be a cause of OSA.

**Figure 3:**
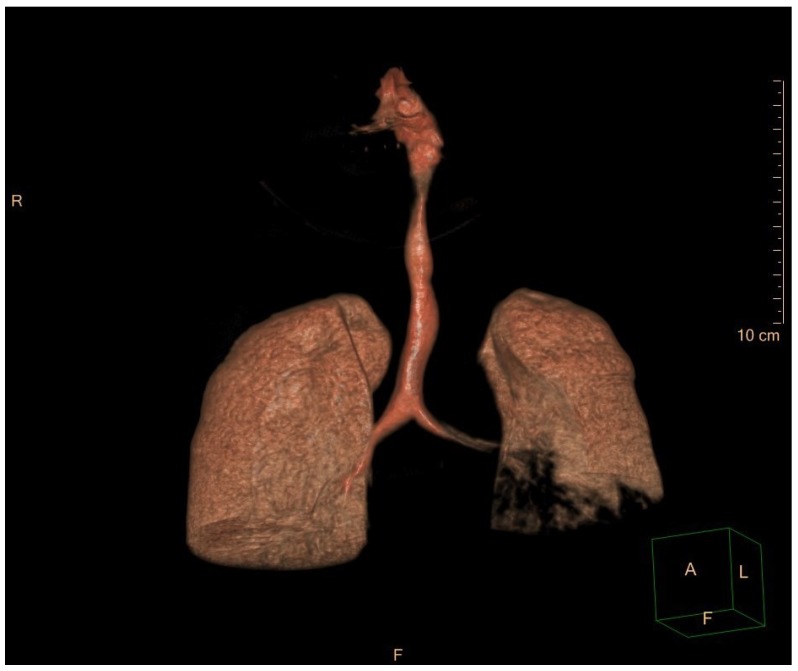
3D CT scan dataset for multiplanar reconstruction shows marked tracheal narrowing and tortuosity.

Thickening of the roof and lateral wall of the nasopharinx and tracheal narrowing, as shown in Figure 3, contributed to the development of severe OSA syndrome in a 12-year old female patient affected by MPS VI or Maroteaux–Lamy syndrome (OMIM 253200), homozygous mutation IVS6-2A>G (c.1214A>G) in *ARSB* gene.

Patients with this syndrome usually have narrow nasal passages, enlarged tongues, short and hypomobile necks, hypoplastic mandibles, increased adenoid and tonsillar size, engorged soft tissues of the nasopharynx, narrowed trachea with thickened epiglottis and vocal cords causing upper and lower airways obstructive-restrictive lung disease [18]. Our patient was first diagnosed with OSA syndrome at three years of age, and therefore underwent adenotonsillectomy. Adenoidectomy was repeated again one year later because of relapse of adenoid hypertrophy. When 12 years old, she was diagnosed again with OSA (apnea-hypopnea index 39.5 events/h) with severe nocturnal oxygenation impairment (oxygen desaturation index 39.8 events/h) and snoring index 25.8% of total registration time. During a sleep endoscopy, the patient developed a severe obstruction of high airways with oxygen desaturation till 50%. After oral intubation, supraglottic airways were explored, adenoid hypertrophy was excluded, and overabundant mucosal folds were observed. Nocturnal NIV was started (inspiratory positive airway pressure (IPAP) 12 cm H_2_O, expiratory positive airway pressure (EPAP) 6 cm H_2_O and respiratory frequency of 18 acts/minute), obtaining a reduction in the number and duration of apneas, representing an improvement of nocturnal oxygen saturation and quality of life. This case report well shows how complicated the clinical course of patients with MPS and ML may be, with recurrence of the same clinical manifestations caused by multiple factors.

Furthermore, these patients may also develop hypoventilation, which may arise from respiratory muscle weakness secondary to neurological cord deficit due to myelopathy, particularly in MPS IVA [12]. In a study by Hendriksz et al., long-term ERT with elosulfase alfa in MPS IVA improved up to week 72 and then stabilized maximum voluntary ventilation (MVV) [19]. Forced vital capacity (FVC) and forced expiratory volume in the first second (FEV1) increased continuously over 120 weeks in a statistically significant way, while untreated patients faced progressive deterioration [19].

The abovementioned obstructive and restrictive impairments, as well as hypoventilation, present functional consequences including respiratory infections, airways compromission during anesthesia or sedation (challenging intubation or extubation) and cor pulmonale [16].

In this scenario, a multidisciplinary approach is of paramount importance in reducing morbidity and mortality.

All MPS/ML patients should be monitored by a pulmonologist with PFTs and polysomnography (PSG) every 6 to 12 months, especially before anesthesia and if respiratory failure is detected [5]. In the case of anesthesia, a bronchoscopy can also provide valuable anatomical information [3,7,20].

Spirometry is recommended as a routine and reliable method for early detection of respiratory impairments—in particular FVC, which can be used to evaluate restrictive lung disease, and MVV, which measures airways resistance and respiratory muscle strength [21]. However, conventional spirometry to evaluate pulmonary function is too challenging in uncooperative patients, such as very young children, and wheelchair-bound patients. In these cases, non-invasive pulmonary tests, including impulse oscillatory system, pneumotachography and plethysmography are preferred [10].

Kubaski et al. performed non-invasive PFTs in Morquio syndrome and showed how these patients have functionally normal lungs, but narrow upper airways and tracheal anatomical abnormalities [10]. Bronchoscopy can show tracheomalacia and narrowing of the trachea due to infiltration of the submucosa and tracheal cartilage. Airway imaging is also useful to better understand the cause of clinical manifestations; cervical and thoracic multi-detector computed tomography (CT) scans with 3D airways reconstruction should be performed periodically, Figure 3 [22].

Cardiological abnormalities, including valvular diseases, arrhythmias, cardiomyopathies and coronary artery disease, also have an impact on lung function. In fact, progression of pulmonary hypertension with cor pulmonale and congestive heart failure may be insidious; therefore, regular cardiological monitoring is recommended [23].

ERT decreases urinary GAG levels and hepatomegaly by decreasing organ storage, but it does not pass the blood-brain barrier or cardiac valves [7,24]. Improvement and long-term stabilization of pulmonary function have been described in all types of MPS in which ERT is available (MPS I, II, IVA, VI and VII) [3,25]. The effect of ERT is due to a variety of mechanisms associated with a decrease in GAG storage, including improvement of joint mobility, endurance and respiratory muscle strength, liver volume reduction and reduced local inflammation [3,25].

Hematopoietic stem cell transplantation (HSCT) can prevent many clinical manifestations, but only if it is performed early in life (before 2 years of age). The purpose of HSCT is the replacement of the hematopoietic compartment with cells that produce normal enzymes derived from the donor. The process of recruiting macrophages and microglia, especially into the nervous system, and their subsequent activation may actually hasten neurodegeneration in these LSD [26]. The success of HSCT depends on the severity of cardiopulmonary and neurological involvement: hepatosplenomegaly, upper airway obstruction with OSA and cardiac manifestations, with the exception of valvular and skeletal deformities, can be improved. A recent multicenter review highlighted the results of HSCT in 217 MPS-IH patients; overnight hypoxia was still observed after transplantation, one patient received tracheotomy for respiratory support, and eight patients required overnight continuous positive airway pressure [7,27].

In summary, the combined effect of lung parenchyma, airway and chest wall abnormalities may lead to respiratory failure in MPS and ML patients, often leading to a poor prognosis beginning in childhood [16,28]. A good awareness and knowledge of these diseases and the association with other systemic symptoms may lead to an early diagnosis, which is essential to prevent complications and ensure therapy [29]. Each patient is unique, and the treatment must be tailored to improve the quality of life of patients and their families.

### 2.2. Molecular Pathways Involved

The storage of GAG/oligosaccharides plays a fundamental role in the pathogenesis of MPS and ML, and it also seems to be the promotor of secondary events that are triggered by the accumulation of undegraded substrates that significantly contribute to complex pathogenetic cascades. Storage of secondary substrates unrelated to the defective enzyme, abnormal composition of membranes, aberrant fusion and intracellular trafficking of vesicles, membranes and membrane proteins, autophagy impairment, alteration of signaling pathways, oxidative stress, abnormalities of calcium homeostasis are only some of the altered pathways involved in the pathogenesis of the disease [30,31]. The dysfunction of each of these pathways impacts and influences the others with an intricate interplay, thus contributing to the complexity of the pathological, functional and phenotypic manifestations of MPS and ML.

## 3. Pompe Disease

Pompe disease, also known as acid alpha-glucosidase (GAA) or acid maltase deficiency or glycogen storage disease type II (GSD II), is a recessive metabolic disorder characterized by accumulation of glycogen within the lysosomes in all tissues.

Pompe disease has a prevalence that varies between populations from 1:40,000 to 1:146,000 and is generally classified as either early- (infantile, classic) or late-onset (non-classic) [32]. The clinical spectrum varies broadly, with significant differences existing in age of onset, rate of disease progression and overall clinical phenotypes.

Classic infantile-onset Pompe disease is a severe disease, in which GAA activity is absent or markedly reduced, leading to death, usually within the first year of life.

Late-onset Pompe disease (LOPD) typically has a later onset during adulthood, because of a higher residual GAA activity, and it usually presents with difficulties in deambulation (proximal limb-girdle myopathy) and respiratory involvement. However, a wide spectrum of phenotypic manifestations has been reported, including bulbar muscle involvement, osteoporosis, scoliosis, rigid spine syndrome, SDB, small-fiber neuropathy, sensorineural hearing loss, cerebral and intracranial aneurysms, cardiac hypertrophy, abnormal cardiac rhythm, impaired gastric function and gastrointestinal motility, lower urinary tract and anal sphincter involvement, pain, and fatigue [33].

### 3.1. Type of Respiratory Involvement

LOPD may cause a progressive respiratory muscle impairment with worsening pulmonary symptoms and respiratory failure. Signs of chronic respiratory failure and decreased sleep quality (fatigue, daytime sleepiness, headaches), and decreased vital capacity (VC) and diaphragm strength may emerge prior to involvement of the extremities. Furthermore, respiratory and abdominal wall muscle weakness leads to ineffective cough and subsequent impairment in airway protection and secretion clearance [33].

Several imaging studies have shown the high prevalence of muscular involvement in Pompe disease [34,35]. Gaeta et al. studied eleven patients with Pompe disease using magnetic resonance imaging (MRI) and PFTs [34]. MRI data confirmed that development of respiratory failure in LOPD is mainly due to diaphragmatic weakness with sparing of the antero-posterior chest expansion related to the activity of the intercostal muscles. Furthermore, in eight out of ten patients, respiratory muscle involvement affected not only the diaphragm, but also the abdominal wall muscles, specifically the internal obliques and rectus abdominis [34]. Wens et al., in a study on spirometer-controlled MRI during maximum inspiration and expiration in ten patients, showed similar results, with smaller cranial-caudal length ratios evident in patients affected by diaphragmatic weakness [35].

Diaphragmatic ultrasound can be used to assess diaphragm weakness and paralysis in Pompe disease patients. Interestingly, a Portuguese study conducted on 18 patients with a diagnosis of diaphragmatic paralysis of unknown etiology revealed that 3 patient out of 18 were affected by LOPD, as per results of dried blood test for GAA activity and confirmed by serum quantification and sequencing. Thus, this study suggests to suspect Pompe disease in all patients with diaphragmatic paralysis of unknown etiology after exclusion of the most common causes.

PFTs are crucial to the evaluation of respiratory muscle weakness. Measurements of FVC both in the seated and supine positions should be made whenever possible, regardless of the presence of respiratory symptoms, because patients may have a normal seated FVC, and the presence of diaphragm weakness can be missed if supine FVC is not measured [36]. A postural drop in VC of more than 25% from the sitting to the supine position is an indicator of diaphragmatic weakness with high sensitivity (79%) and specificity (90%) in various neuromuscular diseases. Other useful measurements for inspiratory muscle weakness detection are maximum inspiratory pressure (MIP) and sniff nasal inspiratory pressure (SNIP).

Impaired coughing effectiveness can be evaluated with use of maximum expiratory pressure (MEP) or peak cough flow (PCF). Values in the range of 50–60 cm H_2_O for the former are consistent with production of a satisfactory cough, while values under and 4.25 L/sec for the latter can help in identifying patients at risk for complications related to impaired clearance of pulmonary airway secretions [37].

Respiratory muscle weakness also contributes to SDB, and to its progression to nocturnal hypoventilation. The diaphragm, rather than the upper airways, is suggested to be the origin of SDB. Characteristics that may differentiate between diaphragmatic weakness and OSA include absence of apneas, lack of correlation with body mass index (BMI), almost exclusive occurrence in REM sleep, and a shift from hypopneas to prolonged hypoventilatory phases directly associated with the restriction worsening. Nocturnal hypoventilation manifests initially with nocturnal hypercapnia and eventually with daytime respiratory failure. Sleep disturbances and non-restorative sleep contribute to physical and mental exhaustion, with symptoms including reduced sleep quality, daytime sleepiness, and fatigue [33]. Detection of SDB is based on PSG, but PFTs (specifically VC) are also useful; patients showing the highest decrease in VC demonstrate more severe hypoventilation and hemoglobin desaturation during sleep [36]. An upright inspiratory VC of less than 40% of the predicted value was shown to be strongly associated with nocturnal hypoventilation in adult Pompe disease patients (sensitivity 80%, specificity 93%) [37].

Long-term LOPD prognosis was ameliorated by the introduction of long-term ERT, which stabilizes pulmonary function and may delay the requirement for mechanical ventilation (MV). Furthermore, MV itself, together with cough assistance and prompt chest infection management, improve patients’ prognosis [38], Table 2.

However, respiratory failure remains a major cause of death [33]. NIV has been shown to correct alveolar hypoventilation and alleviate sleep-related symptoms in patients with both infantile-onset and juvenile/adult-onset Pompe disease [38]. Mellies et al. prospectively applied NIV to eight patients with LOPD and respiratory failure with nocturnal hypoxemia and daytime hypercapnia secondary to severe restrictive lung disease. During a mean (SD) follow-up time of 34 ± 17 months, patients showed oxygen saturation normalization during sleep and improvement in respiratory symptoms and daily partial pressure of carbon dioxide in arterial blood (PaCO_2_), despite further decrease over time of VC and inspiratory muscle strength [39]. Another study on 15 LOPD patients investigated immediate and long-term effects of NIV on sleep and nocturnal ventilation [40]. NIV led to significant improvement of ventilation and oxygenation in the first night of treatment. Follow-up sleep studies revealed stable normoxia and normocapnia without deterioration of sleep outcomes for up to 40 months [40].

Boentert et al., in a recent review on the diagnosis and management of LOPD, suggested using general criteria from guidelines on chronic respiratory failure due to neuromuscular disease [41] to initiate NIV in LOPD patients. Patients with sleep-related symptoms and/or evidence of respiratory muscles weakness should be evaluated with daytime arterial blood gas (ABG) analysis and PSG in order to identify nocturnal hypoventilation and SDB [38]. Ventilation mode, ventilator settings and interfaces should be personalized, humidification should always be offered, and titration of ventilator settings and treatment evaluation should be performed using PSG and PaCO_2_ monitoring. For routine follow-up, serum bicarbonate may be sufficient if NIV is used regularly and patient comfort is good, while ABG and PSG should be performed if serum bicarbonate is elevated or in case of patient discomfort, recurring symptoms of SDB, or marked progression of respiratory muscle weakness [38].

In 2006, ERT with human recombinant acid alpha-glucosidase (Myozyme) became available for all patients in Europe and the USA. LOPD patients treated with ERT showed improvement or stabilization of skeletal muscle strength and respiratory function and prolonged survival. However, the magnitude of the therapeutic response varies among individual patients [42].

Schoser et al. reviewed the long-term efficacy of ERT with regard to survival, motor, and respiratory function in patients with LOPD in relation to the natural progression of the disease. Their analyses showed that ERT reduced the risk of mortality to close to a fifth of that experienced in the natural course of LOPD. Furthermore, they found that patients on treatment demonstrated improved % FVC within the first few months, followed by a gradual return to baseline [43].

In 2017, van der Ploeg et al. published a European consensus for starting and stopping ERT in LOPD patients [42]. The authors suggested starting the therapy in symptomatic patients, keeping in mind that a better clinical status is related to a better response to treatment. Furthermore, the authors suggest not to treat asymptomatic patients, defined as absence of both skeletal muscle weakness assessed by muscle strength tests or impairments in daily living, and respiratory involvement, assessed through PFTs. Follow-up should be performed every 6 months in the first year, and once per year thereafter, in an attempt to identify disease progression early and to start ERT in a timely fashion. Patients with advanced disease (e.g., those who are wheelchair bound and/or ventilator dependent) seem to benefit from ERT, and the therapy should be started unless patients have another significant advanced life-threatening illness or virtually no remaining skeletal or respiratory muscle function. As for duration of the treatment, a period of two years is suggested, after which the effect of treatment should be evaluated. An improvement or stabilization in motor and/or respiratory function suggests that the treatment has a positive effect and should be continued. If the patient shows a substantial deterioration in both motor and respiratory functions, stopping treatment should be considered [42].

Patients’ outcomes depend on many different factors, including age of onset, severity of organ involvement, rate of disease progression, time of initiation and response to ERT.

### 3.2. Molecular Pathways Involved

The GAA gene on chromosome 17q25.3 synthesizes acid alpha-glucosidase, which is a lysosomal enzyme catalyzing alpha 1,4 and alpha 1,6 linkages of lysosomal glycogen. Mutations in the GAA gene, inherited with an autosomal recessive pattern, lead to unstable mRNA, translating into a deficient or null product. The resulting defect in the lysosomal GAA enzyme affects lysosomal-mediated degradation of glycogen. Thus, glycogen accumulates inside lysosomes of skeletal and smooth muscle cells, hepatocytes, endothelial cells, and central nervous system neurons [44].

The progressive accumulation of intralysosomal glycogen leads to lysosomal membranes rupture, causing leakage of hydrolytic material into the cytoplasm with impairment of the muscle contractile unit [45]. The subsequent saturation of autophagic pathways leads to further damage of muscle cells [46].

The diaphragm is the respiratory muscle most frequently involved in the disease, and its dysfunction is not only attributed to myopathic changes, but also to accumulation of glycogen in cervical anterior horn cells leading to alterations of both phrenic nerve fibers and neuromuscular junctions [38].

## 4. Niemann-Pick Disease

Niemann-Pick disease is a group of autosomal recessive disorders associated with splenomegaly, variable neurologic deficits, and the storage of lipids, including sphingomyelin and cholesterol. It is a rare disease without gender predominance with an estimated incidence of 0.4–1 in 100,000 newborns [47].

Three subtypes of the disease are described: Niemann-Pick disease type A and Niemann-Pick disease type B are allelic disorders caused by mutations in the sphingomyelin phosphodiesterase-1 (*SMPD1*) gene, characterized by a primary deficiency of acid sphingomyelinase activity. Niemann-Pick disease type A is the severe early-onset form, whereas type B is the less severe late-onset form. Niemann-Pick disease type C (NPC) is caused by mutations of the NPC1 and NPC2 genes that result in impaired cellular processing and transport of low-density lipoprotein (LDL)-cholesterol.

### 4.1. Type of Respiratory Involvement

Pulmonary involvement, mainly as interstitial lung disease, occurs in all three types of Niemann-Pick disease, but most frequently in type B, with clinical presentation varying from asymptomatic involvement to respiratory failure [47,48]. When present, respiratory symptoms are generally mild, with recurrent cough, moderate exertional dyspnea, and recurrent respiratory infections. However, both rapidly fatal lung disease and progressive pulmonary disease leading to lung failure have been reported. Niemann-Pick disease is characterized by impaired diffusion capacity for carbon monoxide (DLCO) associated with normal spirometric flow rates and lung volumes [49]. Lung volumes may be preserved, even in patients with advanced Niemann-Pick disease with interstitial infiltrates and severely decreased DLCO [47].

On high-resolution computed tomography (HRCT), ground-glass opacities, mild smooth interlobular septal thickening, and intralobular lines, mainly in the lower lung zones, are typical findings. In addition, crazy paving, cysts or pulmonary centrilobular nodular opacities can rarely be appreciated [47,50].

Bronchoalveolar lavage (BAL) or lung biopsy specimens usually confirm lung involvement showing large multivacuolated histiocytes containing granules that stain deep blue with May-Grunwald-Giemsa stain (Niemann-Pick cells or “sea-blue histiocytes”). Furthermore, on histological samples, diffuse endogenous lipoid pneumonia is the classic pattern with infiltration of the lymphatics, subpleural spaces, alveolar walls, and alveoli with Niemann-Pick cells. Niemann-Pick cells also infiltrate the interstitium, but the lung architecture is generally preserved [51].

Lung involvement has been described in only a few patients with NPC, mostly with NPC2 mutation. Staretz-Chacham et al. described pulmonary involvement in nine NPC patients. The first manifestation reported was recurrent pneumonias, followed by recurrent wheezing episodes and subsequent development of interstitial lung disease with hypoxemic respiratory failure [52].

There are no specific therapies for Niemann-Pick disease. With regard to lung involvement, whole lung lavage was reported to be a relatively effective treatment for type B Niemann-Pick disease, especially in adults [47]. However, the outcome of this rare manifestation is still very variable.

### 4.2. Molecular Pathways Involved

In Niemann-Pick disease, organ damage is due to accumulation of lipid-laden macrophages, the so-called Niemann-Pick cells, in various organs, such as the liver, spleen, bone marrow, central nervous system and lung.

In Niemann-Pick disease type A and B, sphingomyelin accumulates within alveolar macrophages [47]. In Niemann-Pick disease type C, the NPC pathway (composed by the large transmembrane NPC protein 1, and the soluble lysosomal NPC protein 2) is blocked. This pathway has a crucial role in intracellular trafficking of cholesterol, finalized to surfactant production. In this latter form of the disease, cholesterol traffic is disrupted with accumulation of cholesterol within type II pneumocytes and alveolar macrophages [52].

Therefore, in the lung, sphingomyelin or cholesterol-laden Niemann-Pick cells accumulate in the alveolar septa, bronchial walls and pleura, leading to a progressively worsening restrictive lung disease [47,52].

## 5. Gaucher’s Disease

Gaucher’s disease is an autosomal recessive multisystemic lipidosis, characterized by organomegaly, hematologic disease and skeletal involvement. In the general population, its incidence is approximately 1/40,000 to 1/60,000 births, rising to 1/800 in Ashkenazi Jews [53]. According to the presence or absence of neurological involvement, three clinical subtypes have been described.

Type-1 Gaucher’s disease, usually distinguished by the absence of neurological impairment, is the most common form of the disease (prevalence 90%–95% in Europe and North America). Its clinical presentation is variable, ranging from asymptomatic disease throughout life to early-onset forms presenting in childhood.

Type-2 Gaucher’s disease is the rarer subtype and is characterized by early and severe neurological impairment with systemic involvement and hepatosplenomegaly leading to death before the third year of life.

Type-3 Gaucher’s disease, also called juvenile or subacute neurological Gaucher’s disease is characterized by the visceral manifestations described in type-1 Gaucher’s disease, usually combined with oculomotor neurological involvement, which appears before 20 years of age in most cases [53].

### 5.1. Type of Respiratory Involvement

In type-1 Gaucher’s disease, pulmonary involvement is rare and may be characterized by interstitial lung disease that may lead to pulmonary fibrosis, chest wall restriction secondary to spinal deformation, or pulmonary hypertension (Group 5—pulmonary hypertension with unclear and/or multifactorial mechanism [54]) [55]. Interstitial lung involvement occurs when Gaucher cells infiltrate alveolar, interstitial and peribronchial spaces. The abnormal cells can also occlude pulmonary capillaries, perhaps contributing to pulmonary hypertension [56]. Furthermore, hepatopulmonary syndrome is a rare complication that may occur in splenectomized patients or in patients affected by portal hypertension complicating hepatic cirrhosis [57].

There are currently two specific types of treatment for Gaucher’s disease: ERT and SRT. The goal is to treat patients before the onset of irreversible complications, such as lung fibrosis. The principle of ERT is to supply the lacking glucocerebrosidase in the cells and therapy, once started, must generally be administered for life. Specific treatment with ERT (Imiglucerase or Velaglucerase) should be considered for all Type-3 Gaucher’s disease patients, while in Type-1 Gaucher’s disease, the only candidates are those with symptomatic clinical or biological abnormalities. SRT (Miglustat or Eliglustat) aims to reduce excessive cell glucocerebrosides accumulation by decreasing its production. Only Eliglustat is suggested as first-line treatment for patients with Type-1 Gaucher’s disease, while Miglustat is a second-line treatment to be used when ERT is no longer accepted by the patient or cannot be used due to intolerance [17].

Goiten et al. described pulmonary involvement and response to ERT in eight patients (four children and four adults) from their referral clinic, who presented with pulmonary signs and symptoms. After starting ERT, patients reported a decreased incidence of clinical signs of lung disease. Two adults showed improvement in oxygen saturation but worsening of pulmonary hypertension with increased interstitial marking on chest X-ray. The other two adults were functionally stable, and one of them showed apparent improvement on HRCT. In light of these results, the authors suggested a possible clinical benefit from ERT on respiratory involvement. However, in contrast to the dramatic reduction in organomegaly, PFTs or lung architecture showed no normalization. Furthermore, the role of ERT in preventing de novo pulmonary involvement still needs to be determined [58].

In conclusion, the outcome of pulmonary involvement may vary according to patients’ subjective factors not yet identified, severity of the disease and treatment response.

### 5.2. Molecular Pathways Involved

Gaucher’s disease is an autosomal recessive disorder that results from deficiency of a lysosomal enzyme glucocerebrosidase (also known as glucosylceramidase or acid beta-glucosidase, GBA), whose gene is located on chromosome 1q21. Glucocerebrosidase is a glycoprotein enzyme whose major substrate is glucocerebroside, a component of the cell membrane that is widely distributed in organs. In affected patients, the deficiency of glucocerebrosidase leads to accumulation of glucocerebroside and other glycolipids within the lysosomes of macrophages.

The clinical manifestations of Gaucher’s disease result from the accumulation of the lipid-laden macrophages in the spleen, liver, bone marrow, bones and other tissues/organs.

## 6. Fabry Disease

Fabry disease is an inherited X-linked lysosomal storage disease due to the deficiency of the enzyme alpha-galactosidase A that results in the accumulation of glycosphingolipids, particularly globotriaosylceramide (Gb3) or globotriaosylsphingosine (lyso-Gb3), within the lysosomes of a variety of cells. Its prevalence in Europe varies between 1/476,000 in the Netherlands to 1/366,000 in the UK, and men are more severely affected than women [59]. Fabry disease mainly affects the kidney, heart and nervous system, but respiratory involvement, including airway obstruction, interstitial lung disease and increased incidence of OSA, has also been described [60].

### 6.1. Type of Respiratory Involvement

The accumulation of glycosphingolipids was observed in the pneumocytes, in the muciparous goblet cells, in the bronchial ciliate epithelium, in the bronchial smooth muscle cells and in the pulmonary vessels on pulmonary biopsy [61], thanks to electron microscopy [62] and postmortem analysis [63].

Respiratory symptoms, including dyspnea, dry cough and bronchospasm, are often nonspecific since they can also be caused by cardiac involvement [60].

The most common respiratory abnormality observed is obstructive airway disease. The first cases described date back to about 40 years ago [64,65]. Since then, several observations have been reported [66,67,68,69,70].

Obstructive airway disease, defined as FEV1/FVC <70% [71], was found in a percentage of patients ranging from 27% to 36%, according to the different cohorts of Fabry patients [66,68,72]. This prevalence is higher than that observed in the general population, in which it ranges between 18% and 33% [73,74]. Furthermore, when considering early spirometric signs of airway obstruction, such as small-airway obstruction, lung hyperinflation and increased airways resistance, the prevalence among patients with Fabry disease ranges between 37 and 68% [66,67,70,75].

However, not all cases of airway obstruction are secondary to Fabry disease itself: when obstructive ventilatory disorder is detected, other concomitant causes or modifiable factors, such as asthma and cigarette smoking, must be excluded [70].

The effect of the ERT on obstructive airway disease is still matter of debate. Few longitudinal studies have evaluated the progression of respiratory involvement in patients with Fabry disease with and without ERT [66,76,77,78]. The first study by Magage et al. in 2008 followed up 39 Fabry patients who were not candidates for ERT for 2 years [66]. The authors observed that the earliest and most frequent PFT abnormalities detected were small–medium airway obstruction, while FEV1 alteration was observed in more advanced stages of the disease, particularly in male and elderly patients [66]. Odler and colleagues observed a stabilization of PFTs during a 5-year follow-up in five Fabry patients with irreversible airway obstruction who were on ERT [76]. Similar results were observed by Faverio and coauthors in a cohort of six Fabry patients on ERT (with and without airway obstruction at baseline) during a median 4-year follow-up [77]. However, a recent observational study by Franzen et al. on a cohort of 95 Fabry patients showed a FEV1 decline of 29 mL per year, with a significantly higher reduction in patients on ERT [78].

Recently, oral chaperone therapy has become available for Fabry patients with amenable mutations; however, the potential effects of this new treatment on obstructive airway disease still need to be explored.

Therefore, further prospective studies are needed to establish the impact of the specific therapies on obstructive ventilatory impairment in Fabry disease.

No studies have been performed to test the efficacy of bronchodilator agents in patients with Fabry disease and airway obstruction. However, the opinion of the authors of this review is that bronchodilators may be tested in patients with airway obstruction, particularly in those who show a spirometric improvement after bronchodilation test, to evaluate their possible positive effect on respiratory symptoms.

Interstitial lung involvement is anecdotally described in Fabry disease [79,80]. In both the cases reported by Kim et al. and Wang et al., a radiological improvement or stabilization was observed after ERT initiation [79,80]. In the case report by Wang et al., peribronchial fibrosis and smooth muscle hyperplasia was observed at lung biopsy and electron microscopy showed accumulations of globotriasosylceramide in bronchial, arteriolar and endothelial smooth muscle cells [80]. This multicellular involvement can translate into different radiological patterns: ground glass opacities may reflect the alveolar filling by glycosphingolipids, while mosaic attenuation at CT scan may indicate, once again, small-airway disease [81].

Chronic fatigue and excessive daytime sleepiness are common findings in patients with Fabry disease and possible causes of such symptoms are chronic heart and renal failure and central nervous system involvement. However, these symptoms may also occur in patients without severe cardiac, renal or central nervous system impairment [82,83]. These observations paved the way to studies aimed to evaluate the prevalence of OSA in patients with Fabry disease.

Franzen et al. performed a PSG and assessed daytime sleepiness and disease severity through the Epworth Sleepiness Scale and Mainz Severity Score Index, respectively, on 52 patients with Fabry disease. The authors found that 13 patients (25%) had SDB (78% OSA and 22% central sleep apneas) [84]. Talbot et al. also observed SDB in 10 out of 20 patients with Fabry disease who underwent a PSG [85]. A possible explanation of the high prevalence of SDB in Fabry disease is the deposition of glycosphingolipids in the muscles of the upper airways, similarly to the hypothesized genesis of obstructive ventilatory disorders in the lower airways.

In conclusion, in Fabry patients presenting respiratory symptoms, the respiratory etiology, although less frequent than the cardiac one, should be included among the possible differential diagnoses. PFTs and chest X-ray are currently the most sensitive and specific tests to investigate respiratory involvement. Chest CT scan and PSG should be considered only in selected cases when interstitial lung disease or SDB are suspected.

### 6.2. Molecular Pathways Involved

In Fabry disease, target organ damage was considered to be caused by the passive progressive deposition of unwanted materials in the lysosomes, leading to functional changes in cells and tissues [86].

However, lysosomal Gb3 and lyso-Gb3 storage due to alpha-galactosidase A deficiency resulting from mutations in the alpha-galactosidase A gene does not entirely explain the pathogenetic processes. In fact, recent evidence suggests that substrate accumulation activates pro-inflammatory mechanisms triggering a chronic pathogenetic cascade including immunological processes, cytokines activation, reactive oxygen species release and cellular apoptosis [87]. These combined pathological pathways could lead to tissue remodeling, development of fibrosis, and, consequently, organ damage.

## 7. Conclusions

The wide range of clinical manifestations and severity of pulmonary involvement in lysosomal storage disorders highlights the importance of keeping a high level of suspicion for respiratory signs and symptoms in these patients. Diagnostic tests available vary according to the type of disease and patients’ age.

Despite the different molecular pathways involved some clinical manifestations and therapeutic approaches are common among diseases, as summarized in Table 2, suggesting that lysosomal storage and subsequent cellular toxicity are the common endpoint.

Future perspectives imply a better understanding of the pathogenetic mechanisms leading to pulmonary involvement and studies on the effectiveness of the available therapies, still very limited for this organ involvement.

## Figures and Tables

**Table 1:** Keywords used to perform the research.

Lysosomal Storage Diseases (OR Mucopolysaccharidosis OR Mucolipidoses OR Pompe disease OR Niemann-Pick disease OR Gaucher’s disease OR Fabry disease) AND Lung involvement (OR Lung manifestations OR Respiratory involvement OR Respiratory manifestations OR pulmonary involvement OR pulmonary manifestations OR Pulmonary function tests OR restrictive lung disease OR obstructive lung disease OR airway obstruction OR interstitial lung disease OR pulmonary hypertension OR obstructive sleep apnea OR sleep-disordered breathing OR hypoventilation OR muscle weakness OR diaphragmatic weakness OR respiratory failure)

**Table 2:** Therapeutic approaches available in different lysosomal storage diseases.

Current Treatments	Mucopolysaccharidosis	Mucolipidoses	Pompe Disease	Niemann-Pick Disease	Gaucher’s Disease	Fabry Disease
Symptomatic treatments for rhinitis and otitis (nasal wash and decongestions, pressure equalization tubes)	X	X				
Inhaled Beta2-agonists and anticholinergic agents ± inhaled corticosteroids to optimize bronchodilation	X	X				X
Airway clearance techniques, including cough assistance when appropriate	X	X	X			
Prompt chest infections’ management	X	X	X	X	X	X
Non-Invasive Ventilation with positive airway pressure treatment (continuous or bilevel) with or without oxygen supplementation (for obstructive sleep apneas and/or muscle weakness)	X	X	X			
Tracheostomy for tracheal obstruction and/or invasive mechanical ventilation	X	X	X			
Tonsillectomy and adenoidectomy	X	X				
Tracheal (-vascular) reconstructive surgery	X	X				
Stents or laser excision of tracheal lesions	X	X				
Whole lung lavage				X		
Enzyme Replacement Therapy	X	X	X		X	X
Substrate Reduction Therapy					X	
Hematopoietic Stem Cell transplantation	X	X				
Smoking cessation	X	X	X	X	X	X

## Abbreviations

ABG	Arterial blood gases
BAL	Bronchoalveolar lavage
BMI	Body mass index
CT	Computed tomography
DLCO	Diffusion capacity for carbon monoxide
EPAP	Expiratory positive airway pressure
ERT	Enzyme replacement therapy
FEV1	Forced expiratory volume in the first second
FVC	Forced vital capacity
GAA	Acid alpha-glucosidase
GAG	Glycosaminoglycans
Gb3	Globotriaosylceramide
GBA	Acid beta-glucosidase or glucosylceramidase
GSD II	Glycogen storage disease type II
HRCT	High-resolution computed tomography
HSCT	Hematopietic stem-cell transplantation
IPAP	Inspiratory positive airway pressure
LDL	Low-density lipoprotein
LOPD	Late-onset Pompe disease
LSD	Lysosomal storage diseases
Lyso-Gb3	Globotriaosylsphingosine
MEP	Maximum expiratory pressure
MIP	Maximum inspiratory pressure
ML	Mucolipidosis
MPS	Mucopolysaccharidosis
MRI	Magnetic resonance imaging
MV	Mechanical ventilation
MVV	Maximum voluntary ventilation
NIV	Non-invasive ventilation
NPC	Niemann-Pick disease type C
OSA	Obstructive sleep apnea
PaCO_2_	Partial pressure of carbon dioxide in arterial blood
PCF	Peak cough flow
PFTs	Pulmonary function tests
PSG	Polysomnography
REM	Rapid eye movement
SDB	Sleep-disordered breathing
SNIP	Sniff nasal inspiratory pressure
SRT	Substrate reduction therapy
VC	Vital capacity
